# A Cross-Sectional Study on the Diet and Nutritional Status of Adolescent Girls in Zambézia Province, Mozambique (the ZANE Study): Design, Methods, and Population Characteristics

**DOI:** 10.2196/resprot.3109

**Published:** 2014-03-05

**Authors:** Liisa Korkalo, Riitta Freese, Lourdes Fidalgo, Kerry Selvester, Carina Ismael, Marja Mutanen

**Affiliations:** ^1^Division of NutritionDepartment of Food and Environmental SciencesUniversity of HelsinkiHelsinkiFinland; ^2^Food Security and Nutrition Association (ANSA)MaputoMozambique

**Keywords:** methods, cross-sectional study, adolescent girls, diet, nutritional status, Mozambique, sub-Saharan Africa

## Abstract

**Background:**

There is very little published work on dietary intake and nutritional status of Mozambicans. We conducted a population-based cross-sectional study on the diet and nutritional status of adolescent girls in different types of communities in Zambézia Province, Central Mozambique, in two distinct seasons.

**Objective:**

The purpose of this paper is to present the design, methods, and study population characteristics of the Estudo do Estado Nutricional e da Dieta em Raparigas Adolescentes na Zambézia (the ZANE Study).

**Methods:**

Data was collected in January-February 2010 ("hunger season") and in May-June 2010 ("harvest season"). A total of 551 girls in the age group 14-19 years old were recruited from one urban area and two districts (district towns and rural villages). The study protocol included a background interview, a 24-hour dietary recall interview, a food frequency questionnaire, anthropometric measurements, bioimpedance, hemoglobin measurement, and venous blood, urine, buccal cell, and fecal sampling.

**Results:**

Adolescent motherhood was common in all study regions. Stunting prevalence for the total study population as a weighted percentage was 17.8% (95/549; 95% CI 14.3-22.0) with no regional differences. Overweight was found mainly in the urban area where the prevalence was 12.6% (20/159; 95% CI 7.5-17.6), thinness was rare. There were regional differences in the prevalence of malaria parasitemia and intestinal helminth infestation, but not human immunodeficiency virus.

**Conclusions:**

The fully analyzed data from the ZANE Study will yield results useful for setting priorities in nutrition policy and further research on the diet and nutritional status in Mozambique and other countries with similar nutritional problems.

**Trial Registration:**

ClinicalTrials.gov: NCT01944891; http://www.clinicaltrials.gov/ct2/show/NCT01944891 (Archived by WebCite at http://www.webcitation.org/6L9OUrsq8).

## Introduction

### Undernutrition in Mozambique

Mozambique is one of the least developed countries in the world with almost 60% of the population living below the international poverty line [1]. Chronic undernutrition is a severe public health problem in Mozambique, as evidenced by a stunting rate of 42.6% in children below the age of 5 years [2]. The majority of the population is rural [3]. Poor households suffer from both chronic and seasonal food insecurity characterized by reducing the number of meals during times of shortage [4]. Problems of widespread poverty and food insecurity, in addition to other factors such as lack of education, poor water and sanitation systems, and frequent infections, are recognized as underlying contributors to undernutrition [5].

While the problem of undernutrition persists in Mozambique and much of Southern Africa, the region is also experiencing the phenomenon of nutrition transition [6]. This epidemiological transition is characterized by three conditions: (1) increased consumption of foods such as energy-dense snack foods, carbonated sweetened beverages, added sugar and fat; (2) decreased physical activity; and (3) increased rates of overweight and noncommunicable diseases [6]. Typically in East and Southern Africa, the increase in overweight and obesity prevalence has been higher in urban than in rural areas [7]. However, in an analysis of nutrition transition indicators [8], Mozambique was shown to be at an earlier stage in the nutrition transition process than some other sub-Saharan African countries, for example neighboring South Africa.

### Lack of Published Work

There is a lack of published work on dietary intake and biochemical indicators of the nutritional status of Mozambicans, especially in age groups other than children below 5 years in age. There are two studies, one for women [9] and one for children [10], that found low median or mean proportions of dietary energy from protein (10% to 11%) and fat (7% to 11%). A review of dietary studies of women in other Sub-Saharan African countries found that intakes of folate, zinc, and iron are often below recommended nutrient intakes [11]. The previous study in Mozambican women mentioned above, conducted during the season of high mango consumption, showed that the prevalence of adequate intake for riboflavin, niacin, folate, vitamin B_12_, calcium, and iron was below 50% [9]. It has been shown, in Mozambique [12] and elsewhere in sub-Saharan Africa [13,14] that energy and micronutrient intakes may fluctuate substantially from season to season. Therefore, studies that cover more than one season are needed in order to have a more complete picture of the diet.

With regard to biochemical indicators of nutritional status, anemia is known to be a severe problem in Mozambique—the Demographic and Health Survey 2011 found that 54% of girls and women of 15 to 49 years of age were anemic [2]. Furthermore, vitamin A and iodine deficiencies are prevalent among children [15,16]. Beyond these indicators, little is known on micronutrient deficiencies in the Mozambican population.

### The ZANE Study

In the *Estudo do Estado Nutricional e da Dieta em Raparigas Adolescentes na Zambézia* (ZANE Study), our aim was to explore the diet and nutritional status of adolescent girls in Zambézia Province, Mozambique, in two seasons and in different types of communities. Adolescent girls, as a group, are vulnerable to the adverse effects of malnutrition. However, this age group is less studied than young children. The ZANE Study was conducted in 2010 and was a population-based, cross-sectional study with an observational design. In this paper, we describe the study design, fieldwork methods, and characteristics of the study population. We also present prevalence estimates for household hunger, overweight, thinness, stunting, human immunodeficiency virus (HIV), malaria parasitemia, and intestinal helminths. Results on the diet and the biochemical indicators of nutritional status are subject of forthcoming publications.

## Methods

### Setting

The Bioethical Committee of the Ministry of Health in Mozambique approved the study plan.

**Figure 1:**
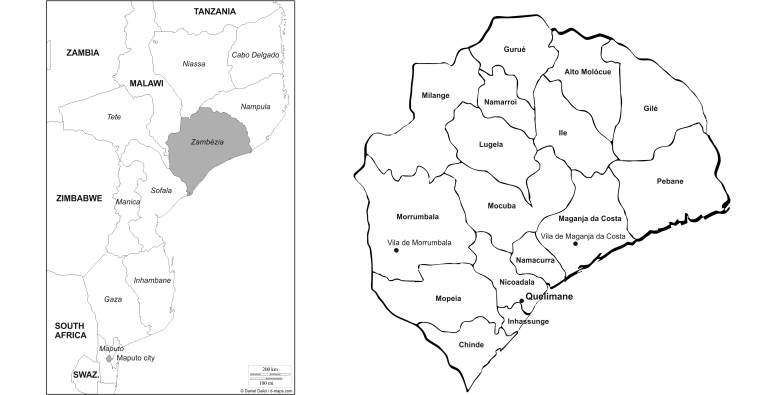
Map of Zambézia Province. The original maps are from d-maps.com [18] and Instituto Nacional de Estatística (Anuário Estatístico, Província da Zambézia, 2003).

Zambézia Province with 3.89 million inhabitants [17], located in Central Mozambique (Figure 1 shows this location) [18] was selected as the study area after discussions with Mozambican authorities. The provincial health directorate decided upon the provincial capital (Quelimane) and two districts (Maganja da Costa and Morrumbala) as the specific study regions.

Quelimane City has 196,000 inhabitants [17]. Economic activities in the city include fishing, coconut production, and sea salt production in addition to the informal commerce of fresh food, clothes, and basic household items. Maganja da Costa is a coastal district with a population of 280,000 inhabitants and Morrumbala is an inland district with 362,000 inhabitants [17]. The main source of livelihood in the rural areas is subsistence agriculture and fishing. The most important crops cultivated are cassava, maize, sweet potato, and rice. Other important crops include cowpeas, pigeon peas, sorghum, groundnuts, cashew nuts, and coconut [19,20]. The rainy season in Zambézia usually lasts from October-November to March-April. The “hunger season” usually lasts from October-November to February-March [21,22], and the main harvest of maize, cowpeas, and groundnuts is in April-May [22].

### Sampling and Recruitment

Adolescent girls in the age range of 15 to 18 years old, residing in the selected study regions, constituted the target study population. The sample size of 600 girls was determined based on the resources available. A sample size of 100 girls from each region per season was considered feasible. The number of primary sampling units (PSUs) selected from each region was dependent on practical considerations. The aim was to have an equal sample size from each PSU.

For purposes of the study, the two districts were divided into district towns and rural areas. The sampling was carried out in five areas: (1) Quelimane City, (2) the district town of Maganja da Costa (*Vila de Maganja da Costa*), (3) rural villages in Maganja da Costa, (4) the district town of Morrumbala (*Vila de Morrumbala*), and (5) rural villages in Morrumbala. The sampling was carried out in two stages in each of the areas, where the first stage involved selection of the PSUs, for example, neighborhoods (*bairros*) or villages, and the second stage involved the selection of households within each PSU.

In the city and two district towns, data from the 2007 Census provided information on the population sizes of the neighborhoods. One neighborhood with a small population size was removed from the sample framework. In the two rural areas, locally obtained maps provided information on the villages. Villages located within a 45 to 60 minute drive from the study centers (health centers and hospitals) were considered as PSUs. There were no available listings of households in the PSUs.

The study was undertaken in two seasons—January-February, 2010 (“hunger season”) and May-June, 2010 (“harvest season”). The PSUs were selected using probability proportional to size (in the city and the district towns) or random sampling (in rural areas). In the city, sampling was carried out only in the catchment areas of three health centers out of the nine health centers across the city for practical reasons. The same PSUs were used in both seasons. For the second stage, the recruiters sketched a map of each selected PSU with the help of local leaders. The recruiters were instructed to follow a recruitment plan which included randomly selected starting points on the map and randomly selected directions to walk in order to chart the households. The recruiters were instructed to select households using preset intervals.

Girls in the target age range, who were able to visit the study center and did not have any significant illness preventing participation, were considered eligible. Eligible girls along with their parent, husband, or guardian were given an informed consent letter. The purpose of the study, the measurements and tests taken, the voluntary nature of participation, and the right to refuse to participate in any part of the study were also explained orally. The girl signed the informed consent form. If she was under 18 years of age, a parent, husband, or guardian also signed the form. If the girl or the adult was illiterate, a fingerprint or a signature of a witness indicated informed consent. The date of birth was verified by checking identity cards whenever possible. Alternatively, the time of birth was estimated with the help of family members. No incentives to participate in the study were given. The recruited girls were invited to come to the study center on the following day, and participants who lived far from the study center were provided with transport.

**Figure 2:**
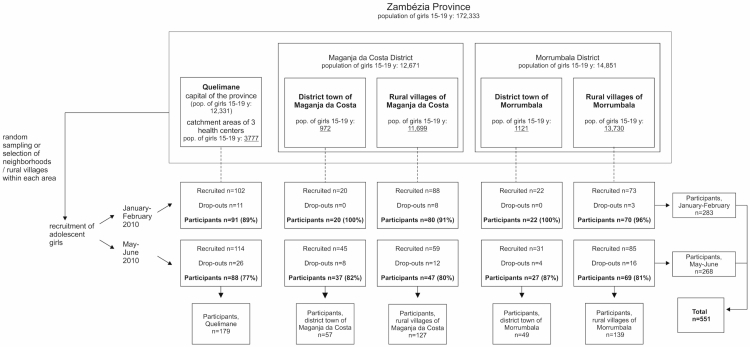
Implemented sampling design. The population figures are from the 2007 Census. y=years.

The total number of eligible girls refusing to participate is not known. Reasons for refusing to participate included refusal of parents to give permission to be part of the study, not having the time, and fear of the hospital or of blood sampling. A total of 639 girls agreed to participate, of whom 551 actually participated in the study (Figure 2 shows the sampling design). There were twenty-seven 14-year-olds and four 19-year-olds that were included in the final sample due to mistakes in checking the age during recruitment. However, their data was kept in the analysis since according to the World Health Organization (WHO) definition, persons 10 to 19-years-old are considered adolescents [23].

A total of 143 participants were assigned to a subsample for an additional 24-hour dietary recall interview in January-February 2010 and two additional 24-hour recalls in May-June 2010. There was a total of four 24-hour recalls for the participants of this subsample. Because most of them did not have a precise address or a phone number, recontacting the participants of this subsample proved to be difficult. In January-February 2010, we were able to recontact 109 of the subsample participants for the second interview. In May-June 2010, 96 of those were successfully recontacted for a third interview, and 84 of those for a fourth interview. The additional 24-hour recalls were conducted on nonconsecutive days.

There were practical difficulties obtaining all information needed for multi-stage cluster sampling, for example, detailed maps with demarcation of all PSUs were not available and the population size of eligible girls in all PSUs was not known. Furthermore, recruiting an equal sample size from each selected PSU turned out not to be successful. In some cases, nonrandomly selected PSUs had to be included. For example, a selected village had to be replaced by another one when the leaders of the village were not supportive of the study. Taking into consideration all of these factors, sampling weights were calculated using the total population sizes of 15-19-year-old girls in the five areas from the 2007 Census. The population sizes used for calculating the sampling weights are shown (underlined) in Figure 2. The sampling weights were used when data from the different study areas were pooled for analysis.

### Background Interview

All forms and questionnaires used in the study were in Portuguese. However, if the participant was not comfortable in Portuguese, the questions were explained by field workers and appropriate interpreters in a language understood by the participant. The background questionnaire included questions about the characteristics of the participant and her household, for example, schooling, mother tongue, marital status, the number of children, pregnancy and breastfeeding status, self-reported health status, usage of mosquito net, cigarette and alcohol use, exposure to media (television, radio, newspaper), ownership of livestock and other assets, sanitation, and the type of housing. Asking each participant to read out a short test sentence in Portuguese tested literacy; being literate was defined as the ability to read the whole sentence. The background interview also included a Portuguese version of Household Food Insecurity Access Scale (HFIAS) questions [24,25]. These questions were used to calculate a more recently developed measure called Household Hunger Scale (HHS) [26], which is based on selected questions from the HFIAS. Locally recruited interviewers with varying backgrounds conducted the background interviews.

### Food Frequency Questionnaire

A 7-day food frequency questionnaire (FFQ) was designed specifically for this study. The list of foods included in the FFQ was based on the experience accumulated during preliminary fieldwork and questionnaires on dietary diversity or food consumption from previously published [25] and unpublished surveys conducted in Mozambique. The FFQ was peer reviewed by a team of Mozambican nutritionists. It was not validated. The FFQ included a list of 37 commonly consumed foods, additional lines for other items belonging to selected food groups, and a line for dietary supplements. The interviewer requested participants to estimate the number of times they had consumed any of those food items during the previous 7 days. The response categories were: none; 1 to 2; 3 to 4; 5 to 6; 7; and, more than 7 times. With regards to oil and margarine, the respondent was asked to specify the type, or preferably brand name, in addition to the consumption frequency. The same interviewers who conducted the background interviews filled in the FFQs.

### Twenty-Four-Hour Dietary Recall Interview

For the 24-hour dietary recall interview, the participant was asked to recall and report on the main activities that she had engaged in during the previous day. She was then asked to report all the food and beverages she had consumed during that period. The information gathered from the participant on the daily activities was used for probing. With regard to composite dishes, the participant was asked to describe all the ingredients in the dish. For the data collection period of May-June 2010, a separate question about the use of iodized salt was added to the form.

**Figure 3:**
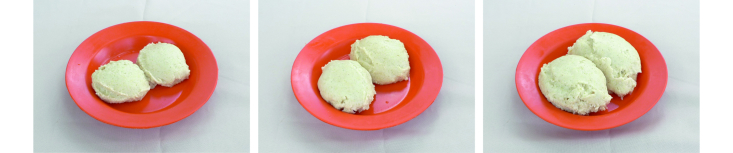
Example of food photographs used in portion size estimation: 270 g, 408 g, and 610 g of thick maize porridge.

A set of food photographs was shown to the participant to help her with the estimation of portion sizes (Figure 3 shows an example of these photographs). Experiences gained from a validation study [27] were used when producing the set of photographs; the new photographs were scaled down from life-sized by approximately 5%, and the presentation of the foods was slightly modified. A plate (same as in the photographs), cup, spoon, and two different ladles that were typical of the location were also used in the portion size estimations. Finnish nutrition students and Mozambican nutrition technicians conducted the 24-hour dietary recall interviews. The Finnish interviewers spoke English, which was interpreted by local interpreters. In some cases, the Mozambican interviewers or interpreters did not have a common language with the participant and, when needed, additional *ad hoc* interpreters were used.

To calculate nutrient intake, a Mozambican food composition database was compiled. The majority of the food composition data was taken from the United States Department of Agriculture National Nutrient Database for Standard Reference [28]. Other literature sources included composition tables of other African countries and journal articles. Samples of 35 foods were also collected during preparatory fieldwork in 2008-2009 and analyzed in Finland. A detailed list of sources of data for each food item is available on the Internet [29]. The food composition database is freely available for those interested in conducting future work on dietary intake in Mozambique [30].

Recipe data was collected during visits to local households by recording the weight of each ingredient and the prepared dish. Of the collected recipes, 24 were relevant and directly useable, or could be modified by changing some ingredients. In addition, yield factors (to adjust for weight changes during cooking) for a few foods that include only one main ingredient in addition to water, for example, thick maize porridge, were collected. Some yield factors were also adapted from other sources [31,32]. Nutrient retention factors (to adjust for nutrient losses during cooking) were taken from the literature [33]. NutriSurvey [34] was used for data entry and analysis. The main ingredients of composite dishes were modified in the data entry as reported by each participant.

### Anthropometric and Other Measurements

For the most part, anthropometric measurements were carried out according to the WHO technical instructions [35]. The participants were barefoot and dressed in their normal light clothing. Their heights were measured by a portable statiometer. If a participant’s hairdo prevented exact measurement, the thickness of the hairdo was measured by a dipstick and subtracted from the measured height. Weight was measured using a digital scale.

The participants’ waist circumference, right upper arm length, and mid-upper arm circumference were measured after marking of the anatomical sites [35,36]. Total arm length was measured as the distance between the acromion and the tip of the middle finger. Inelastic circumference measuring tape was used and the recordings were taken to the nearest 0.1 cm. The mean of two measurements was used.

The participants’ triceps and subscapular skinfold measurements were measured using a Harpenden Skinfold Caliper (Baty International, UK) according to the instructions of the manufacturer. The anatomical sites were marked [36] and three measurements of each skinfold were taken without releasing the grasp of the skinfold. The final value was the mean of the three measurements. If the three measurements varied by more than 1 mm, the measurements were repeated.

Hand-to-foot bioimpedance (Bodystat 1500 DMM, Bodystat Ltd, UK) was used to measure body composition of the participants. Bioimpedance measurements were taken in the standing position [37] instead of the usual lying position due to limitations of the study center facilities.

A member of the field team used a digital automatic blood pressure monitor (Omron M6, Omron Healthcare, Japan) to measure the blood pressure and pulse of the participants. Only one measurement was taken as most participants were unfamiliar with the procedure and the protocol did not allow time for them to become accustomed to it.

Participants’ forearm muscle strength measurements were taken using a hydraulic hand dynamometer (SH5001, Saehan Corporation). The participant was encouraged to grip the handle with all her strength. There were two successive measurements that were taken for both hands. The result used in the study is the higher reading from each hand.

### Laboratory Procedures and Biological Samples

The recruiters gave participants a fecal sample container and instructed them to deposit the sample at home. The laboratory technicians performed a direct wet mount with saline and examined the specimen (n=353) for the presence of helminth eggs or larvae under a microscope.

During the study center visit, the participants were asked to provide a spot urine sample. The samples were tested for pregnancy (Insight-HCG, Tulip Diagnostics, India; or Onsite HCG Combo Rapid Test, CTK Biotech, USA) and aliquots were frozen for further analyses. Although it was not part of the study protocol, unprompted, the laboratory technicians of Morrumbala District checked suspect (samples with dark brown or red coloration) urine samples for the presence of *Schistosoma haematobium* (*S haematobium*) by microscoping the sediment that was obtained after centrifugation. The positive cases (n=12) were provided with the appropriate medication.

Buccal cells were obtained by scraping the inside of the cheek of each participant with a new toothbrush [38]. Bottled drinking water was used for rinsing the mouth and the toothbrush. The samples were transferred to 15 ml conical bottom centrifuge tubes for centrifugation at 4500 rpm (1900 x g) for 10 minutes. The supernatant was discarded and the sediment was suspended in 1 ml 0.9% saline and stored in -15 to -20 °C freezer.

Venous blood samples (n=515) were taken from the antecubital vein using 21 g needles in serum tubes (10 ml) and 3 ml tubes containing potassium ethylenediaminetetraacetic acid (EDTA) (K3EDTA) (BD Vacutainer, Becton Dickinson International, Belgium). The EDTA-blood sample was centrifuged to plasma at 4500 rpm (1700 x g) for 10 minutes and aliquots were frozen and stored at -15 to -20 °C. The serum tubes were allowed to stand at room temperature (22-36 °C) for 30 minutes, and then centrifuged and frozen as described above. The study team purchased freezers to ensure adequate cold storage in the study centers. The samples were transported to Maputo in a portable freezer by car where they were packed in dry ice and shipped to Finland. In Finland, they were stored at -70 °C.

EDTA-blood was used for hemoglobin determination with HemoCue Hb 301 System (HemoCue AB, Sweden). The presence of malaria (*Plasmodium falciparum*) antigens (histidine-rich protein II, or HRP-II, antigens) was determined by using a one-step test (SD BIOLINE Malaria Ag P.f, Standard Diagnostics, Korea). Antibodies to HIV-1/HIV-2 were detected principally using Determine HIV-1/2 (Inverness Medical Japan, Japan) test kits, and positive results were confirmed with another test (Uni-Gold HIV, Trinity Biotech, Ireland). In some cases, capillary blood was used for hemoglobin, malaria, and HIV tests when EDTA-blood was not available. HIV testing was permitted by 372 of the participants.

Drops of EDTA-blood were applied to the sample collection area of Whatman 903 Protein Saver Cards, dried, and packed in Ziploc storage bags with desiccant packs. The cards were kept at room temperature until delivered to Finland, where they were stored at -20 ˚C.

A local nurse gave the results of the pregnancy, hemoglobin, malaria, and intestinal helminth infestation tests to the participants, and provided iron supplements for those with hemoglobin levels below the local cut-off, 110 g/l. Antihelminthics and malaria treatments were also provided for those participants whose test results indicated these conditions. Participants who tested positive for HIV were referred to the physician who was responsible for the study in a particular region in order to have counseling and subsequent treatment.

### Statistical Analyses

Numbers and proportions of participants for selected background characteristics were computed. It should be noted that pregnancy was defined based on a urine test result or the participant being visibly pregnant. If this information was not available, those who self-reported not being pregnant were coded as not pregnant and those who self-reported being pregnant were coded as missing.

Z-scores for body mass index-for-age (BMI) and height-for-age were calculated using a macro by WHO [39], which uses the WHO 2007 growth reference for school-aged children and adolescents. Participants with a height-for-age z-score < -2 SD were classified as stunted. Nonpregnant participants with a BMI-for-age z-score < -2 or > 1 SD were classified as thin or overweight, respectively.

Prevalence estimates with 95% confidence intervals (CI) were computed for the categories of BMI-for-age and height-for-age z-scores, HHS, HIV, malaria parasitemia, and presence of intestinal helminths. For these analyses, the data from the district towns and rural areas were aggregated in each district in order to present results that are representative at district level. Seasonal differences in each area were examined before combining the two seasons, and regional differences were examined with the seasons combined. Proportions were compared using Pearson's chi-square tests with the Rao and Scott second order correction. Prevalence estimates for the aggregated total study population were also computed for selected variables. Sampling weights, as described above, were used in the analyses. Analyses were carried out using the survey package in R version 3.0.1.

## Results

### Date of Birth

The exact date of birth was known for 75.5% (416/551) of the participants, and for 92.1% (383/416) of those, the recruiters reported having verified the date of birth from an identity card or other official documents. For 24.5% (135/551) of the participants, only the year or year and month of birth were recorded.

### Background Characteristics

The background characteristics (Table 1) showed that literacy rates varied from 11.2% (15/134) in rural Morrumbala to 81.5% (145/178) in Quelimane. There were one in five to almost one in three girls that had already given birth or were pregnant at the time of the study. Sanitation was poor; in the rural areas many households had no toilet at all. The most commonly reported mother languages (or dialects) were Chuabo and Portuguese in Quelimane, Nyaringa in Maganja da Costa District, and Sena in Morrumbala District.

**Table 1:** Characteristics of the study population.

		Quelimane	Maganja da Costa District		Morrumbala District	
			District town	Rural villages	District town	Rural villages
		n=179	n=57	n=127	n=49	n=139
**Age, n (%)**						
	14 years	10 (5.6)	2 (4)	5 (3.9)	1 (2)	9 (6.5)
	15 years	52 (29.1)	19 (33)	45 (35.4)	21 (43)	62 (44.6)
	16 years	39 (21.8)	9 (16)	27 (21.3)	9 (18)	30 (21.6)
	17 years	46 (25.7)	13 (23)	22 (17.3)	7 (14)	18 (12.9)
	18 years	32 (17.9)	13 (23)	27 (21.3)	11 (22)	18 (12.9)
	19 years	0 (0.0)	1 (2)	1 (0.8)	0 (0)	2 (1.4)
	Missing data, n	0	0	0	0	0
**Currently attending school or studying**					
	n (%)	170/177 (96.0)	49/56 (88)	77/127 (60.6)	42/49 (86)	91/134 (67.9)
	Missing data, n	2	1	0	0	5
**Literate** ^a^					
	n (%)	145/178 (81.5)	28/53 (53)	39/124 (31.5)	20/48 (42)	15/134 (11.2)
	Missing data, n	1	4	3	1	5
**Married** ^b^					
	n (%)	10/179 (5.6)	7/56 (13)	30/127 (23.6)	4/49 (8)	40/133 (30.1)
	Missing data, n	0	1	0	0	6
**Currently pregnant** ^c^					
	n (%)	18/178 (10.1)	6/56 (11)	20/125 (16.0)	1/49 (2)	15/135 (11.1)
	Missing data, n	1	1	2	0	4
**Has given birth or is currently pregnant**					
	n (%)	38/176 (21.6)	16/55 (29)	38/125 (30.4)	10/49 (20)	41/133 (30.8)
	Missing data, n	3	2	2	0	6
**Currently breastfeeding**					
	n (%)	9/177 (5.1)	8/55 (15)	9/127 (7.1)	6/49 (12)	23/135 (17.0)
	Missing data, n	2	2	0	0	4
**Type of sanitation, n (%)**						
	No toilet	16/179 (8.9)	2/56 (4)	54/127 (42.5)	2/49 (4)	79/135 (58.5)
	Pit latrine	141/179 (78.8)	53/56 (95)	72/127 (56.7)	47/49 (96)	56/135 (41.5)
	Flush toilet	22/179 (12.3)	1/56 (2)	1/127 (0.8)	0/49 (0)	0/135 (0.0)
	Missing data, n	0	1	0	0	4
**Main source of energy for cooking, n (%)**						
	Firewood	2/178 (1.1)	28/56 (50)	114/127 (89.8)	19/49 (39)	121/136 (89.0)
	Charcoal or coal	175/178 (98.3)	27/56 (48)	13/127 (10.2)	30/49 (61)	15/136 (11.0)
	Electricity or gas	1/178 (0.6)	1/56 (2)	0/127 (0.0)	0/49 (0)	0/136 (0.0)
	Missing data, n	1	1	0	0	3

^a^able to read a full test-sentence in Portuguese

^b^married or traditionally married, includes divorced or separated and widowed

^c^positive—urine test result positive/visibly pregnant; negative—urine test result negative, or if test result not available, self-report of not being pregnant

### Anthropometry

The majority of the girls had a BMI-for-age in the normal range and overweight was found mainly in the city (Table 2). There were eleven participants that were classified as thin. None were obese (>2 SD). The prevalence estimate for stunting among the total study population as a weighted percentage was 17.8% (95/549; 95% CI 14.3-22.0) with no regional differences. There were no seasonal differences in the BMI-for-age and height-for-age categories.

### Household Hunger

There were eleven participants that were classified as living in households suffering from severe hunger according to the HHS. The two categories “moderate” and “severe household hunger” were combined for analysis. Household hunger prevalence estimate for the total study population as a weighted percentage was 17.0% (101/541; 95% CI 13.6-21.1) with no statistically significant regional differences.

**Table 2:** Prevalence estimates (weighted percentages) for categories of height-for-age and BMI-for-age z-scores, and Household Hunger Scale in the study regions^a^.

		Quelimane, n=179	Maganja da Costa District, n=184	Morrumbala District, n=188	*P* ^b^
**Height-for-age z-score**					.329
	normal (≥ -2 SD), n (weighted %)	156/178 (87.6)	149/183 (83.0)	149/188 (80.2)	
	95% CI	82.9-92.4	76.9-89.0	74.1-86.3	
	stunted (< -2 SD), n (weighted %)	22/178 (12.4)	34/183 (17.0)	39/188 (19.8)	
	95% CI	7.6-17.1	11.0-23.1	13.7-25.9	
	Missing data, n	1	1	0	
**BMI-for-age z-score** ^c^					< .001
	normal (-2 to 1 SD), n (weighted %)	136/159 (85.5)	150/154 (98.7)	159/168 (95.5)	
	95% CI	80.2-90.9	96.9-100	92.1-98.8	
	thin or severely thin (< -2 SD), n (weighted %)	3/159 (1.9)	2/154 (1.0)	6/168 (4.0)	
	95% CI	0-4.0	0-2.8	0.7-7.3	
	overweight (> 1 SD), n (weighted %)	20/159 (12.6)	2/154 (0.3)	3/168 (0.5)	
	95% CI	7.5-17.6	0-0.7	0-1.1	
	Missing data or pregnant, n	20	30	20	
**HHS**					.274
	little or no HH, n (weighted %)	137/177 (77.4)	148/179 (81.9)	155/185 (85.4)	
	95% CI	71.4-83.4	75.5-88.2	80.0-90.8	
	moderate or severe HH, n (weighted %)	40/177 (22.6)	31/179 (18.1)	30/185 (14.6)	
	95% CI	16.6-28.6	11.8-24.5	9.2-20.1	
	Missing data, n	2	5	3	

^a^Sampling weights were used. Seasonal differences within each area were tested with chi-square test before pooling data from two seasons and significant differences were found for HHS in Quelimane (Jan-Feb: weighted percentage 30%, 27/90, vs May-June: weighted percentage 15%, 13/87; *P*=.02).

^b^Overall test (chi-square) of regional differences after pooling data from the two seasons. This test was followed by pairwise tests.

^c^In pairwise comparisons, Quelimane differed significantly from Maganja da Costa (*P*<.001) and from Morrumbala District (*P*<.001).

### HIV, Malaria Parasitemia, and Intestinal Helminths

The prevalence estimate for HIV among the total study population as a weighted percentage was 6.0% (29/372; 95% CI 3.8-9.3); there were no differences between regions (Table 3). Of the 510 participants tested, 46 were positive for malaria parasitemia. Of these positive malaria cases, 42 were found in Maganja da Costa.

The prevalence estimate for intestinal helminth infestation among the total study population as a weighted percentage was 11.7% (48/353; 95% CI 8.4-16.1). Significantly higher prevalence of intestinal helminths was found for Quelimane as compared to the districts (Table 3). All positive intestinal helminth cases in Maganja da Costa District were from the rural villages. Nearly all cases of *Ascaris lumbricoides* (*A lumbricoides*) and *Trichuris trichiura* (*T trichiura*) were found in Quelimane*,* whereas all cases of *Strongyloides stercoralis* (*S stercoralis*) were found in the districts. *Ancylostoma duodenale* (*A duodenale*) was found both in the city and in the districts.

**Table 3:** Numbers of positive cases and prevalence estimates (weighted percentages) for HIV, malaria parasitemia, and intestinal helminth infestation in the study regions^a^.

		Quelimane, n=179	Maganja da Costa District, n=184	Morrumbala District, n=188	*P* ^b^
**HIV**					
	n (weighted %)	10/103 (9.7)	10/117 (7.4)	9/152 (4.5)	.32
	95% CI	4.1-15.3	2.0-12.7	1.2-7.9	
	Missing data, n	76	67	36	
**Malaria parasitemia** ^c^	
	n (weighted %)	2/164 (1.2)	42/168 (26.2)	2/178 (0.9)	< .001
	95% CI	0-2.9	18.6-33.7	0-2.3	
	Missing data, n	15	16	10	
**Intestinal helminth infestation** ^d,e^	
	n (weighted %)	28/105 (26.7)	7/138 (6.8)	13/110 (13.1)	.007
	95% CI	18.4-35.0	2.0-11.7	6.4-19.9	
	Missing data, n	74	46	78	

^a^Sampling weights were used. Seasonal differences within each area were tested with chi-square test before pooling data from two seasons and significant differences were found for intestinal helminths in Maganja da Costa (Jan-Feb: weighted percentage 12%, 7/71, vs May-June: weighted percentage 0%, 0/67; *P*=.002) and in Morrumbala (Jan-Feb: weighted percentage 20%, 10/58, vs May-June: weighted percentage 6%, 3/52; *P*=.04).

^b^Overall test, chi-square, of regional differences after pooling data from the two seasons. This test was followed by pairwise tests.

^c^In pairwise comparisons, Quelimane differed significantly from Maganja da Costa (*P*<.001), and Maganja da Costa differed significantly from Morrumbala District (*P*<.001).

^d^In pairwise comparisons, Quelimane differed significantly from Maganja da Costa (*P*<.001) and from Morrumbala District (*P*=.02).

^e^The species identified: *A duodenale*—18 findings, *A lumbricoides*—12 findings, *T trichiura*—10 findings, *S stercoralis*—7 findings, and *S mansoni*—2 findings. One participant was infested with two species.

## Discussion

### Purpose of the Study

We have collected data that will provide in-depth information on the nutritional situation of adolescent girls in Zambézia Province, Central Mozambique. The purpose of this paper is to describe the design and methods of the ZANE Study and to present descriptive results on the characteristics of the study population.

### Food Insecurity, Stunting, and Overweight

The proportion of adolescent girls living in households suffering from at least a moderate level of hunger is markedly lower in our study compared to the 57% [40] found in two previous surveys in other central provinces of Mozambique. This difference could be explained by numerous factors including differences in the geographical area, year and season of data collection, and the fact that in our study the respondents were adolescent girls. In contrast, the age and sex of respondents of the previous surveys were unspecified [25]. HHS measures a severe range of household food insecurity including such experiences as not having any food to eat and going to sleep at night hungry [26]. Thus, even the smaller proportions of households suffering from food insecurity found in our study indicate that this is a problem in the area.

Although thinness was found to be rare, close to one in five of the adolescent girls in our study area were stunted. Previously, a school-based study in the capital city Maputo found that of 2.3% of girls 6 to 18 years of age were stunted [41]. Differences in the sampling and growth reference used prevent detailed comparisons with Maputo girls, but, nevertheless, it is clear that the prevalence of stunting was higher among our population-based sample.

We found that overweight was more common in the city compared to the districts. Although this result is based on cross-sectional data, it could be interpreted as an early sign of nutrition transition in the urban adolescent girls in Central Mozambique. The finding also supports nationally representative data on adults [42], which showed that the proportions of overweight/obese people were higher in urban areas compared to rural areas for both men and women.

### HIV and Intestinal Helminth Infestation

The estimate for HIV prevalence was similar to the national prevalence among 15-19-year-old girls, 9.3% [43]. The reasons for participant refusals for HIV testing were not recorded, but some of the participants who refused were already aware of their HIV-positive status. The prevalence of intestinal helminth infestation in our study was clearly lower compared to the previous study of Augusto et al [44], where the prevalence of soil-transmitted helminth infestation in Zambézia among 7-22-year-olds was found to be as high as 51%. Differences in the prevalence estimates between this study and that of Augusto et al may be due to differences in the methodology and age ranges of the participants. In our study, only 67.5% (372/551) of the participants agreed to be tested for HIV and 64.1% (353/551) provided fecal samples for testing for intestinal helminths, and this may have reduced the accuracy of the prevalence estimates.

### Fieldwork Challenges

Challenges were encountered during the fieldwork. The lack of detailed maps made it difficult to plan the sampling beforehand, and the sampling design had to be adjusted during the fieldwork. Although the field team had strong local representation through the inclusion of district agricultural and health workers, in a few cases building trust with the local community leaders was not successful, and for that reason not all villages initially selected in the sampling were included in the study. The timetable for carrying out the sampling was restricted, and when recruitment was not possible at a chosen location, a village had to be chosen nonrandomly as a replacement in order to ensure the continuity of the daily flow of participants throughout both study periods. Despite these limitations in the sampling design, recruitment of participants was largely successful, and the data provides a good representation of the target population in the areas studied.

### Informed Consent

Informed consent is a critical component of study ethics. It has been shown in other studies that participants may have difficulties understanding all aspects of the informed consent; this applies for both low-income country and high-income country settings [45]. In Uganda, it was shown that the majority of participants in observational studies reported having enough information about the study *per se*, but about one third did not have full knowledge about their right to voluntarily withdraw from the study at any time [46]. The understanding of the informed consent was not assessed in our study, but the fact that there were refusals to participate due to fear of blood sampling suggests that some eligible candidates may not have fully understood their freedom to refuse blood sampling while being free to participate in other parts of the study. On the other hand, many participants refused HIV testing, which is a positive sign that some of the participants did exercise their right to refuse to participate in specific aspects of the study. A number of girls (n=88) who consented to participate did not arrive at the study center (Figure 2). The reason for this remained unknown for the majority, and it is possible that many simply forgot and the study team was not able to find them again. However, some candidates did mention they had changed their mind or that the head of household or community leader refused for them to participate even after informed consent had been given.

### Summary of the Results

To summarize, the results on the characteristics of the study population of the ZANE Study indicate that the girls suffer from deprivation; the regions studied are characterized by poor sanitation facilities, and adolescent girls suffer under the burden of household hunger, infectious diseases, and intestinal helminths. The rate of adolescent motherhood is high in all the study areas, and literacy rates are particularly low in the rural areas. The forthcoming results on dietary intake and nutritional status will be useful for setting priorities in nutrition policy, and for further research on adolescent girls in Mozambique or countries with similar nutritional problems.

## Abbreviations

BMI: body mass index
CI: confidence interval
EDTA: ethylenediaminetetraacetic acid
FFQ: food frequency questionnaire
HFIAS: Household Food Insecurity Access Scale
HHS: Household Hunger Scale
HIV: human immunodeficiency virus
PSU: primary sampling unit
WHO: World Health Organization
ZANE Study: Estudo do Estado Nutricional e da Dieta em Raparigas Adolescentes na Zambézia

## References

[1] UNDP (2013). Human development report 2013.

[2] (2013). Moçambique Inquérito Demográfico e de Saúde 2011.

[3] World Bank (2012). World Bank.

[4] UNICEF (2011). Child poverty and disparities in Mozambique 2010.

[5] (2013). UNICEF.

[6] Vorster HH, Kruger A, Margetts BM (2011). The nutrition transition in Africa: Can it be steered into a more positive direction?. Nutrients.

[7] Popkin BM, Adair LS, Ng SW (2012). Global nutrition transition and the pandemic of obesity in developing countries. Nutr Rev.

[8] Abrahams Z, McHiza Z, Steyn NP (2011). Diet and mortality rates in Sub-Saharan Africa: Stages in the nutrition transition. BMC Public Health.

[9] Arimond M, Wiesmann D, Becquey E, Carriquiry A, Daniels MC, Deitchler M, Fanou-Fogny N, Joseph ML, Kennedy G, Martin-Prevel Y, Torheim LE (2010). Simple food group diversity indicators predict micronutrient adequacy of women's diets in 5 diverse, resource-poor settings. J Nutr.

[10] Low JW, Arimond M, Osman N, Cunguara B, Zano F, Tschirley D (2007). A food-based approach introducing orange-fleshed sweet potatoes increased vitamin A intake and serum retinol concentrations in young children in rural Mozambique. J Nutr.

[11] Torheim LE, Ferguson EL, Penrose K, Arimond M (2010). Women in resource-poor settings are at risk of inadequate intakes of multiple micronutrients. J Nutr.

[12] Rose D, Tschirley D (2003). Predicting dietary intakes with simple food recall information: A case study from rural Mozambique. Eur J Clin Nutr.

[13] Bates CJ, Prentice AM, Paul AA (1994). Seasonal variations in vitamins A, C, riboflavin and folate intakes and status of pregnant and lactating women in a rural Gambian community: Some possible implications. Eur J Clin Nutr.

[14] Nyambose J, Koski KG, Tucker KL (2002). High intra/interindividual variance ratios for energy and nutrient intakes of pregnant women in rural Malawi show that many days are required to estimate usual intake. J Nutr.

[15] Aguayo VM, Kahn S, Ismael C, Meershoek S (2005). Vitamin A deficiency and child mortality in Mozambique. Public Health Nutr.

[16] WHO (2006). WHO global database on iodine deficiency, Mozambique.

[17] Instituto Nacional de Estatística.

[18] d-maps.com.

[19] Ministério da Administração Estatal (2005). Perfil do Distrito de Maganja da Costa, Província da Zambézia.

[20] Ministério da Administração Estatal (2005). Perfil do Distrito de Morrumbala, Província da Zambézia.

[21] (2010). Famine Early Warning Systems Network.

[22] (2010). Famine Early Warning Systems Network.

[23] WHO (1986). Young people’s health-A challenge for society. WHO technical report series No. 731.

[24] Coates J, Swindale A, Bilinsky P (2007). Household food insecurity access scale (HFIAS) for measurement of food access: Indicator guide (v. 3).

[25] FAO FAO food security project GCP/MOZ/079/BEL.

[26] Ballard T, Coates J, Swindale A, Deitchler M (2011). Household hunger scale: Indicator definition and measurement guide.

[27] Korkalo L, Erkkola M, Fidalgo L, Nevalainen J, Mutanen M (2013). Food photographs in portion size estimation among adolescent Mozambican girls. Public Health Nutr.

[28] (2008). USDA.

[29] Korkalo L, Hauta-alus H, Mutanen M (2011). Food composition tables for Mozambique, Version 2.

[30] Department of food and environmental sciences.

[31] Matthews R, Garrison Y (1975). Agriculture handbook No. 102: Food yields summarized by different stages of preparation.

[32] Bognár A (2002). Tables of weight yield of food and retention factors of food constituents for the calculation of nutrition composition of cooked foods (dishes).

[33] USDA (2003). USDA table of nutrient retention factors, Release 5 (Release 6 available online).

[34] NutriSurvey.

[35] WHO (1995). Physical status: The use and interpretation of anthropometry. Report of a WHO Expert Committee. Technical Report Series No. 854.

[36] National Health and Nutrition Examination Survey (2007). Anthropometry procedures manual.

[37] Rush EC, Crowley J, Freitas IF, Luke A (2006). Validity of hand-to-foot measurement of bioimpedance: Standing compared with lying position. Obesity (Silver Spring).

[38] London SJ, Xia J, Lehman TA, Yang JH, Granada E, Chunhong L, Dubeau L, Li T, David-Beabes GL, Li Y (2001). Collection of buccal cell DNA in seventh-grade children using water and a toothbrush. Cancer Epidemiol Biomarkers Prev.

[39] World Health Organization Growth reference for 5-19 years. Application tools.

[40] Deitchler M, Ballard T, Swindale A, Coates J (2010). Validation of a measure of household hunger for cross-cultural use.

[41] Prista A, Maia JA, Damasceno A, Beunen G (2003). Anthropometric indicators of nutritional status: Implications for fitness, activity, and health in school-age children and adolescents from Maputo, Mozambique. Am J Clin Nutr.

[42] Gomes A, Damasceno A, Azevedo A, Prista A, Silva-Matos C, Saranga S, Lunet N (2010). Body mass index and waist circumference in Mozambique: Urban/rural gap during epidemiological transition. Obes Rev.

[43] Republic of Mozambique National AIDS Council (2012). GARPR (2012 global AIDS response progress report) for the period 2010-2011, Mozambique.

[44] Augusto G, Nalá R, Casmo V, Sabonete A, Mapaco L, Monteiro J (2009). Geographic distribution and prevalence of schistosomiasis and soil-transmitted helminths among schoolchildren in Mozambique. Am J Trop Med Hyg.

[45] Mandava A, Pace C, Campbell B, Emanuel E, Grady C (2012). The quality of informed consent: Mapping the landscape. A review of empirical data from developing and developed countries. J Med Ethics.

[46] Kiguba R, Kutyabami P, Kiwuwa S, Katabira E, Sewankambo NK (2012). Assessing the quality of informed consent in a resource-limited setting: A cross-sectional study. BMC Med Ethics.

